# Pathology Versus Phrenology

**Published:** 1858-01

**Authors:** 


PATHOLOGY VERSUS PHRENOLOGY.—Dr. Dimmick, of La Salle, Illinois, reports, in the December number of the *North-Western Medical and Surgical Journal*, a case in which there was a loss, by injury and subsequent sloughing, of a large part of the anterior lobes of both hemispheres of the brain, without any perceptible diminution, for the time being, of mental power or activity. The substance of the case is as follows :

A boy, two years and a half old, was kicked on the forehead by a colt ; the frontal bone was fractured from one temporal fossa to the other, and the fragments of bone driven through the membranes into the substance of the brain. The first evening after the accident he was a little nauseated, but the three succeeding days he seemed as well as usual, and the family, not suspecting the extent of the injury, allowed him to play about the house, which he did with his accustomed liveliness. On the fourth day convulsions occurred, and Dr. Dimmick having been called, discovered the true state of the case, and proceeded to remove the depressed bone. This was accompanied with a free discharge of purulent matter and blood, and succeeded by paralysis of the right side. "For two or three days," to use the words of the reporter, "the suppuration and discharge of sloughed portions of the brain was profuse, accompanied at times by copious hemorrhage, so that by about the seventh or eighth day after the accident the anterior portion of the frontal lobes of both hemispheres, as far back, I judged, as the corpus callosum, was entirely gone. The orbital plates were laid bare. After washing out the cavity, the brain could be seen and felt, presenting an irregular surface or wall, nearly perpendicular to the base of the skull, and yet, to my surprise, he retained his mental faculties apparently unimpaired." He died two weeks subsequently, three weeks from the time of the injury, and "during the greater part of this time he was perfectly rational. In questioning him on points requiring the exercise of comparison, causalty, eventuality, numeration, form, color, etc., I asked him questions that I do not think could ever have occurred to him, and endeavored to confuse and puzzle him ; but his apt replies and his judgment satisfied me that he was more precocious than most children of his age.

"The natural action of the bowels and bladder continued throughout the whole time."

---

REPORTS.—The present number of the Review contains the first of the series of Reports on Medicine, which, as stated in the last number, will hereafter constitute a separate department of the journal.

The Reports on Surgery, Practical Medicine, and Toxicology, are in course of preparation by Drs. E. HARTSHORNE, of Philadelphia, AUSTIN FLINT, sen., of Buffalo, and B. SILLIMAN, jun., of New Haven, respectively. The names of those engaged upon the remaining branches will be announced in due time.

